# How the alcohol industry fought against pregnancy warning labels in France. A press coverage analysis spanning 20 years

**DOI:** 10.3389/fpubh.2022.933164

**Published:** 2022-08-26

**Authors:** Ana Millot, Martina Serra, Karine Gallopel-Morvan

**Affiliations:** ^1^Univ Rennes, EHESP, CNRS, Inserm, Arènes - UMR 6051, RSMS (Recherche sur les Services et Management en Santé) - U 1309, Rennes, France; ^2^EHESP, Institut du Management, Rennes, France

**Keywords:** alcohol industry, lobbying, warnings, labels, pregnancy

## Abstract

**Background:**

Drinking alcohol while pregnant is dangerous for health. To inform on this issue, various countries have adopted pregnancy warning labels on alcoholic beverages, including France since 2007, where wine holds deep cultural consonance. The aim of this research was to analyze the arguments put forward by the alcohol industry (producers, distributors, wholesalers, allied industries, trade associations, social aspects and public relations organizations, councilors who publicly defend wine-sector interests) *via* the press in France: (1) in 2007 when pregnancy warnings were first implemented, and (2) in 2018 when larger pregnancy warnings to increase visibility were proposed but not adopted.

**Methods:**

We used documentary method to analyze the arguments advanced by the alcohol industry in mainstream (national, regional and specialized) press in France from 2000 to 2020, using the Europresse documentary database. Quantitative analysis (number and trend curve of articles, mapping *alcohol-industry* actors who spoke in the press) and inductive thematic content analysis (analytical framework of the arguments identified) using NVivo software were carried out.

**Results:**

We found a total of 559 relevant press articles in the database, of which 85 were included in the analysis. Peaks in number of publications were found to coincide with the warning label implementation and with the expansion-project schedule. A large majority of the arguments promoted by the alcohol industry contested the pregnancy warnings measure (very few were in favor). They argued that (1) pregnancy warnings were a questionable measure (e.g., ineffective, or the pictogram clearly links alcohol to mortality), (2) pregnancy warnings would have counterproductive effects (on women and the wider economy), (3) better alternatives exist (e.g., targeted prevention programs, prevention by health professionals). A large majority of the actors who spoke in the press came from the winegrowing sector.

**Conclusion:**

This study fills a gap in the Anglosphere research on lobbying against alcohol warnings by analyzing lobbyists' arguments over a 20-year period covering both failed and successful industry lobbying. New findings have emerged that are likely related to the wine-oriented culture of France. In order to counter the alcohol lobbying practices we conclude with a number of public health recommendations.

## Introduction

Drinking alcohol while pregnant is dangerous for health. Fetal alcohol syndrome (FAS) is the most severe form of alcohol use-related damage, and is responsible for physical, intellectual, cognitive and behavioral impairments in the child (1, 2). Europe consistently registers the highest levels of alcohol use during pregnancy in the world, estimated at 25.2%, and the highest prevalence of FAS, at 37.4 cases per 10,000 people (3, 4). In France, where this research takes place, alcohol use during pregnancy is a major public health issue, as an estimated 27% of mothers drink alcohol and the prevalence of FAS is 41.4 per 10,000 people (3). To tackle this issue, the French authorities have introduced three main measures: an annual mass media campaign under the banner “no alcohol while pregnant,” consumption screening and prevention tools for health professionals, and pregnancy warning labels on alcoholic beverages. This paper deals with the on-container warnings measure, which the World Health Organization (WHO) advocates as inexpensive for governments to deploy (5) and effective at informing the public (6, 7). Warnings targeted at women have been compulsory since 2007 in France (8), where producers are to label all alcohol containers with a warning text (stating “Consumption of alcoholic beverages during pregnancy, even in small quantities, can have serious consequences for the health of the child”) or a prohibition sign-like pictogram representing a pregnant woman drinking alcohol crossed out with a red line (see Appendix 1). In practice, most beverages carry the pictogram that is barely visible at an average size of 0.4 cm (9), often placed at the back, embedded in other on-beverage marketing (10).

Equivalent warnings to inform on health have been adopted in only very few countries, such as Australia, New Zealand, Turkey, and the Republic of Moldova (7, 11). One reason of this weak label implementation in the world may be strong lobbying by the alcohol industry (AI) against this measure (7, 12). In Australia for instance, the AI has managed to delay the introduction of a new larger warnings that target pregnant women by 3 years (13, 14).

In France, two research studies stressed that the pictogram currently displayed on containers is poorly noticed (15, 16) and thus ineffective in grabbing attention and informing people (10). As a result, in 2018 an evolution was proposed as part of the 2018–2022 “National Mobilization against Addictions” plan. This evolution in format was designed to improve the visibility of the pictogram by increasing its size and imposing a color or contrast (17). This change met with strong opposition from the AI (18) and has so far not been adopted.

Research is needed to better understand the lobbying activities of the AI first against adoption of the French health warning label (where lobbying failed to block adoption of the labels in 2007) and during the recent warning expansion project (which lobbying managed to block).

There is scant research into lobbying on alcohol warnings in general (12, 13, 19–24) and only four studies on strategies against warnings that target pregnant women (12, 13, 23, 24). Analysis of these strategies found that the AI claimed such health warnings (1) are ineffective, because there is no evidence that pregnant women stop drinking; (2) should be replaced by alternative measures (information and educational programs); (3) are unneeded, because women already know the risks of consuming alcohol; (4) will have negative unintended consequences (create guilt and anxiety among pregnant women); (5) restrict international trade and create added costs for producers; (6) are illegal because they curtail the AI's freedom of speech and there is no legislative authority to compel warnings on alcoholic beverages.

The aim of this research is to bring insight into the AI's lobbying against warnings aimed at pregnant women by analyzing the arguments advanced by the AI in the French press from 2000 to 2020 in an effort to block original implementation of the warnings (2007) and the later warning label expansion project (2018).

Our research makes several contributions. It brings fundamental insight to the limited literature on lobbying against alcohol warnings in general and those aimed at pregnant women in particular. The AI lobbies against the development of effective alcohol control policies around the world (25, 26), so it is important to analyze the strategies it employs in order to better understand them. The analysis proposed here spans a period of 20 years in France, which covers a period that includes the original adoption of alcohol warnings (2007) and the later warning label expansion project (2018) that was not adopted. Analyzing differences and shifts in the AI's arguments may explain why this health measure got implemented in 2007 and why its subsequent expansion got blocked. Another theoretical contribution is to enrich the sets of arguments identified in the extant literature on Anglosphere countries (13, 23) and to propose an analytical framework that may be better adapted to countries like France that have long and deeply embedded ties with alcohol in general and wine in particular. France offers a specific case-setting regarding alcohol and drinking culture, for five reasons: (1) alcohol is widely consumed in France compared to other countries (27): on average, in 2017, the French consume 11.7 L of pure alcohol per year per capita (28); (2) cultural acceptance of wine is very high in French society (29, 30); (3) the “French paradox”, a myth—strongly criticized by the scientific community (4)—asserting that moderate consumption of red wine is good for health remains deeply embedded (30); (4) the economic dimension is important: in 2020, France was the second-largest producer and third-largest exporter of wine worldwide (31); (5) the AI has strong connections with President Emmanuel Macron (32), who was elected “Personality of the Year 2022” by the *Revue du Vin de France* (“French Wine Review” in English) for “his constant commitment to wine and its culture,” an award that he came to collect in person (33, 34).

Beyond these theoretical contributions, our research is also useful for NGOs and health advocates campaigning to raise awareness of the AI's lobbying against health measures and for countries that are planning to introduce their own alcohol warnings (7) or want to maintain or increase the effectiveness of alcohol warnings in place. The discussion section provides recommendations, based on our findings, on how to deal with and counter lobbying by the AI.

## Materials and methods

A qualitative analysis of media documents was conducted to identify the arguments advanced by the AI on French alcohol warnings targeted at pregnant women. The AI is understood to mean “a multi-national business complex that includes not only producers of beer, wine, and distilled spirits but also a large network of distributors, wholesalers, and related industries”(35). Trade associations that promote alcohol producers' interests and “social aspects and public relations organizations” (SAPROs) are thus considered part of the AI (4), along with elected representatives of wine-producing regions or parliamentary groups identified in previous research that publicly defend wine-growing interests within the French government (36, 37).

Press articles were analyzed from the 2000–2020 period in order to identify the full spectrum of discourses (intensity and content) during this relatively long period and arguments used prior to the warnings adopted in 2007 (period 1) until 2 years after 2018, the year that the pictogram expansion project that never got implemented (period 2).

### Data collection

The mainstream press is a good tool for analyzing industry lobbying and arguments (20), as media coverage can influence and frame public debate, public opinion and policy outcomes, especially when governments attempt to implement restrictive measures to improve public health (20, 38, 39).

Here we used the Europresse documentary database, which provides access to a large number of leading print-press sources (dailies, weeklies) ranging from newspapers, magazines, journals and online news content to press releases and newswires (general national and regional press, and specialized press) (40).

The search terms queries in Europresse were “(pictogram OR logo OR labelling) AND (pregnant woman OR pregnancy) AND (alcohol^*^ OR bottle)” (in French: “(pictogramme OU logo OU étiquetage) ET (femme enceinte OU grossesse) ET (alcool^*^ OU bouteille)”).

**Figure 1:**
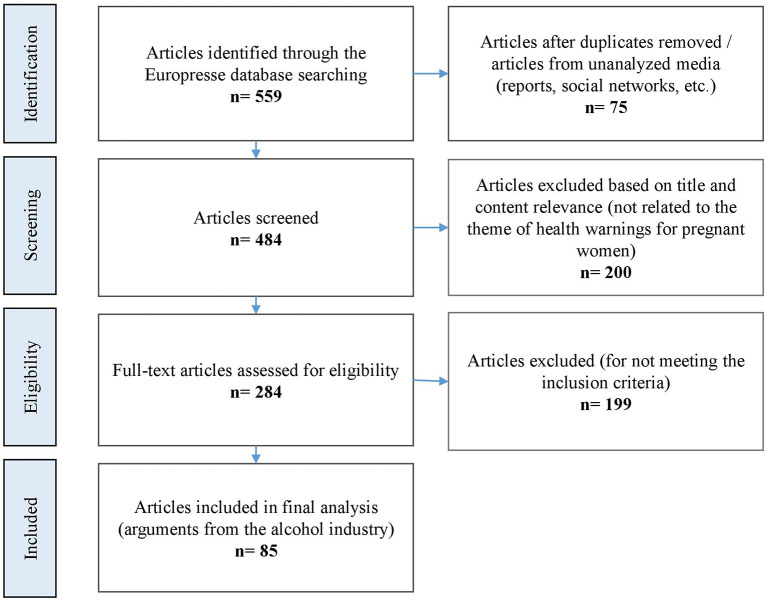
Flow chart of the inclusion process for press articles identified by searching the Europresse database.

Polling the database returned 559 articles: 75 were excluded after deleting duplicates and articles from non-analyzed media (social media, reports, etc.); 200 were excluded based on title and content relevance (i.e., they were unrelated to the theme of warnings for pregnant women); 199 were rejected because they did not express the point of view of the AI (neutral articles or articles expressing the point of view of other actors, mostly NGOs). A total of 85 press articles met the inclusion criteria (articles in which the AI expressed itself or was cited on the issue of warnings aimed at pregnant women) and were included in the final analysis (see Figure 1 and Appendix 2).

### Data analysis

A quantitative analysis was first carried out in order to identify the number and trend-curve of articles covering the research theme over the 2000–2020 period.

The profiles of the AI actors who spoke on the issue were identified. In each press article, the names of the interviewees expressing their views, their company (if mentioned) and their alcohol sector were coded. They were then classified into the following categories: wine, spirit, beer, SAPROs, alcohol sector (in general, when the actors / sector / company were not specified) and other (only one: a printer associated to the AI because he printed labels for alcoholic beverages) (see Appendix 3).

An inductive method was used to analyze the data without “trying to fit the data to pre-existing concepts or ideas from theory” (41). Because it was the first French analysis of the lobbying of the AI against warnings, and because the French context toward alcohol is very specific (e.g., importance of wine in French culture, high level of alcohol consumption), an inductive approach was preferred compared to the use of Anglo-Saxon frameworks suggested previously to analyse the lobbying of the AI against warnings (12, 13, 23). This approach provides detachment from the existing literature and contexts that are different from those of French culture.

A thematic content analysis was then conducted to map content and topics across the data by identifying key themes (41). This analysis consists of an initial reading of all press articles. For each article, the argument(s) used by the AI were identified (e.g., “Ineffectiveness of the measure” or “Counterproductive effects on the economy”) and then grouped into categories of arguments (e.g., “Pregnancy warning labels are a questionable measure” or “The warning would have counterproductive effects”). Once the analytical framework was finalized to highlight the content of the AI's arguments, the occurrence of the same category of arguments was counted.

To conduct the analysis, a researcher independently carried out a manual coding of all press articles. A second researcher coded also independently this material using NVivo12 qualitative research data analysis software. The two analyses were compared, and if any divergences appeared, a team meeting with a third researcher was held to reach a consensus.

## Results

### Pattern of change in the press articles published over the years studied

**Figure 2:**
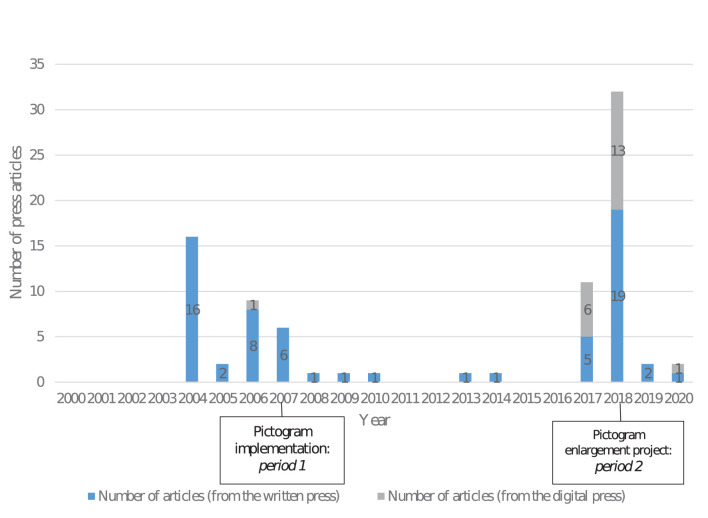
Analyzed press articles published between 2000 and 2020, found in the Europresse database.

A majority of the 85 press articles included in the final analysis (see Appendix 2) came from the written press (only 21 from online press). The number of articles published differed by year, with less articles published around the original implementation in 2007 (period 1: 38 press articles) than around the time of the pictogram expansion project in 2018 (period 2: 47 press articles) (see Figure 2). Both periods coincided with peaks in the number of publications, which is evidence that the AI response to a public health proposal is to react *via* the mainstream press. The rise in the number of articles between 2007 and 2018 may be due to the development of the online press in 2018 compared to 2007 (19 articles were published through the online press in 2018 compared to 1 in 2007) and/or to a professionalization of the lobbying of the AI between the two periods (the AI may use more frequently in 2018 the press as an indirect tool of lobbying). It is also interesting to highlight a drop of articles after 2018 (higher than in 2007). It may be explained by a favorable political context at that time for the AI, especially for the wine industry with which the French President Emmanuel Macron has very close ties (details to come in the discussion section).

### Identification and evolution of who spoke *via* press articles

A large majority of the AI actors who spoke in the press came from the vine and wine sector (93 times) followed by the broader “alcohol” sector (when no specific sector was cited; 14 times) and SAPROs (8 times). Note, however, that the spirits sector (3 times), the beer sector (3 times), and another agent (a printer: 1 time) also voiced opinions *via* the press (see Appendix 3).

**Figure 3:**
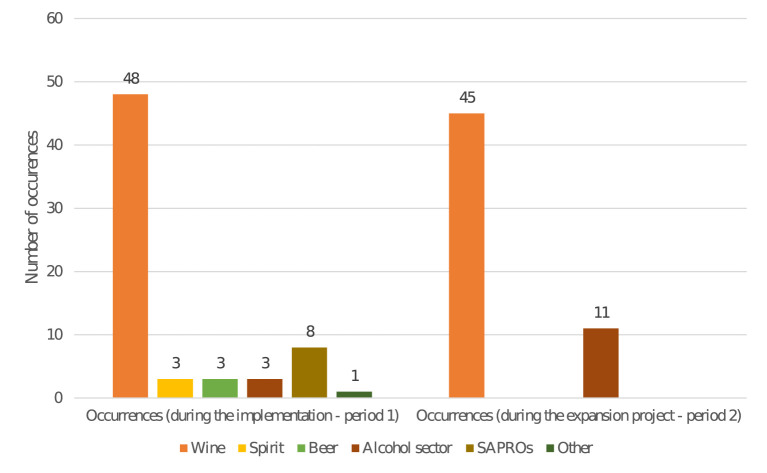
Evolution of the actors who spoke in the media.

Between period 1 and period 2, the number of wine-sector actors using press remained stable, at 48 vs. 45 occurrences (see Figure 3 and Appendix 3), whereas other voices had a weaker and more isolated presence. This could be explained by the fact that the wine sector is well-perceived in France, and is increasingly becoming the front group for all the alcohol actors in the media (36). An identical phenomenon was observed during the lobbying against the Evin Law of marketing regulation in France: the winegrowers were a very visible front group to fight against the law compared to other alcohol actors (36).

### Arguments advanced by the alcohol industry in French media

**Figure 4:**
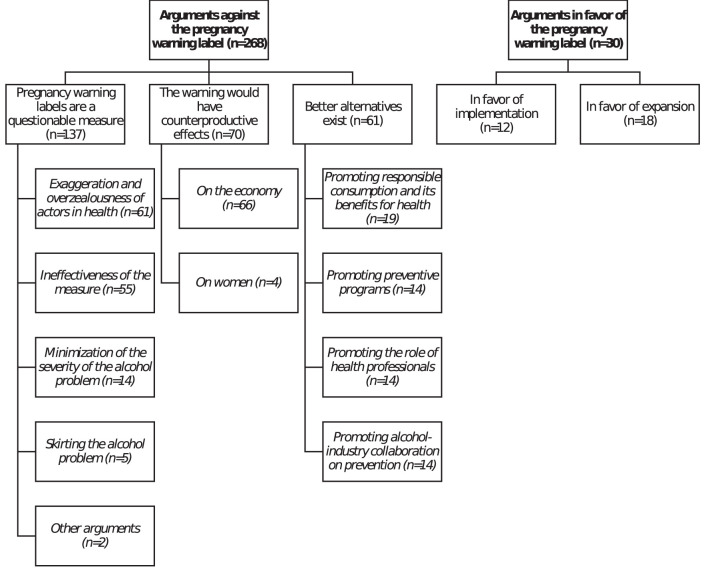
Taxonomy of alcohol-industry arguments.

The majority of the arguments used by the AI from 2000 to 2020 were raised against the original introduction and subsequent evolution of the warning (268 occurrences). There was nevertheless a small minority of AI arguments in favor of the measure (30 occurrences) (see Figure 4 for an overview of arguments and Appendix 4 for details on the numbers of occurrences for each arguments through time). Different sub-categories of arguments emerged from the analysis and are described below.

### Arguments against the French pregnancy warning label

Our analysis suggested there were three categories of arguments against the measure: (1) pregnancy warning labels are a questionable measure, (2) pregnancy warning labels would have counterproductive effects, and (3) better alternatives exist. These categories are outlined below, with examples provided.

#### Pregnancy warning labels are a questionable measure

##### Exaggeration and overzealousness of actors in health (61 occurrences)

The pictogram clearly links alcohol to mortality was one of the main arguments advanced by the AI (28 occurrences), essentially in period 2 (26 occurrences vs. 2 occurrences on period 1):

“*The sector is opposed to what it publicly describes as a ‘deadly pictogram'*” (“Comment le lobby de l'alcool sape toute prévention prônant l'abstinence,” *Le Monde website*, 2020)

The AI claims that this measure is driven by hygiene-first logic, i.e., dictated by the medical perspective (12 occurrences). This was the argument most used during period 2 (10 occurrences vs. 2 during period 1):

“*the winegrowers fear that this enlarged pictogram will lead to a ‘hygiene-first' logic […] where the ultimate form would be a bottle similar to the plain tobacco packaging*” (“Des viticulteurs bordelais entrent en guerre contre le logo femme,” *L'Express website*, 2017)

It also claims that this measure only serves to reassure the health authorities (7 occurrences, of which 5 during period 2):

“*Does the reminder on wine bottles serve any other purpose than to reassure and hypocritically relieve the health authorities of all liability?*” (“Attention, vivre est nuisible à votre santé,” *Le Bien Public*, 2018)

To prove this point, the AI signals that France is one of the only countries to implement the pictogram, which they see as further evidence that the measure is exaggerated (5 occurrences, only during period 1):

“*This constraint is a feature specific to France and is not used in other European countries*” (“Des viticultrices à l'Assemblée,” *Sud Ouest*, 2010)

Finally, it was also mentioned that the measure was disproportionate (3 occurrences) and would open the floodgates to more virulent messages (3 occurrences, only during period 1), such as those for tobacco (“alcohol kills”), arguing that alcohol should not be treated in the same way as tobacco (3 occurrences):

“*And the media coverage of it is odious. Soon, we will see ‘alcohol kills' on labels of good Burgundy wine. Just like on cigarette packets*” (“La filière viticole se sent attaquée, le milieu médical se dit sceptique,” *Le Journal de Saône-et-Loire*, 2004)

“*It is even a ‘total idiocy' that will ‘further fuel the idea that wine is a dangerous product like tobacco'*” (“Déconseillé aux femmes enceintes: un vigneron affiche la couleur,” *AFP Infos Economiques*, 2004)

##### Ineffectiveness of the measure (55 occurrences)

The AI also put forward the ineffectiveness of the warning, arguing that the measure is ineffective in changing behaviors among pregnant women (26 occurrences, 14 occurrences in period 1 vs. 12 in period 2):

“*Women who are addicted to alcohol will continue to drink, just as smokers continue to smoke despite the warnings displayed on tobacco packages. The rest [of the women] already know not to drink during pregnancy*.” (“Le message pour les femmes enceintes n'inquiète pas la filière,” *Le Journal de Saône-et-Loire*, 2007)

“*To date, no comprehensive study has been produced to demonstrate the effectiveness of this measure*.” (“Désaccords autour d'un logo,” *Emballages magazine.com*, 2018)

This ineffectiveness is explained by the poor design and content of label. The AI claims that the warning is not precise enough and could thus create confusion (such as the belief that wine in the presence of the pictogram would be a contraceptive) (2 occurrences, in period 1), and is poorly crafted (1 occurrence, in period 1):

“*When our Chinese customers saw it, they thought our wine was a contraceptive…*” (“Pression sur l'étiquette,” *Sud Ouest*, 2009)

“*Yves d'Amécourt [an elected representative] is outraged by the ‘starkness' of the pictogram, as according to him, ‘what could be more beautiful than a pregnant woman?'*” (“D'Amécourt et l'esthétique de la femme enceinte,” *Sud Ouest*, 2007)

The AI also adds that it is unreadable because container labels are already overloaded with information (11 occurrences, 7 occurrences in period 2):

“*For the Vignerons Indépendants [professional association of independent winegrowers], it is yet another feature to fit on already overcrowded label*” (“Naissance difficile de l'étiquetage préventif des boissons alcoolisées,” *Les Echos*, 2006)

Pregnancy warning labels are touted as ineffective because they are perceived as a “cosmetic” measure (8 occurrences, all during period 2): according to the AI, pregnancy warning labels are considered as superficial and therefore useless. AI actors also consider this measure as inappropriate (7 occurrences) to fight against alcoholism among pregnant women:

“*winegrowers deplore a ‘primarily cosmetic measure'*” (“Non au logo agrandi pour femmes enceintes,” *L'Union*, 2017)

“*The measure is an ‘inadequate response to a real public health issue'*” (“Non au logo agrandi pour femmes enceintes,” *L'Union*, 2017)

##### Minimization of the severity of the alcohol problem (14 occurrences)

The AI minimizes the severity of the issues tied to alcohol use by arguing that women are already informed and responsible (7 occurrences, mostly in period 1):

“*Question: Do pregnant women know that alcohol is dangerous for their unborn child? Answer: To not know, either they'd have to ignore it on purpose or spend their pregnancy lost in a cave in the woods.”* (“Attention, vivre est nuisible à votre santé,” *Le Bien Public*, 2018)

The AI also claims that wine is not alcohol (or at least not an alcohol like any other) (5 occurrences, of which 4 during period 1):

“*Is wine an alcohol like any other? ‘Wine, consumed in moderation, is part of the traditional French foodways. It has to be kept apart from other alcoholic drinks'*” (“Alcool et femmes enceintes le nouveau logo sur les bouteilles de vin fait polémique chez les vignerons,” *AFP*, 2018)

They also added that alcoholics do not tend use wine (1 occurrence, during period 1) and that FAS remains rare (1 occurrence, during period 1):

“*It is even more ridiculous for wine: I have never considered myself as trading in alcoholism, and in any case, alcoholics are not loyal customers of the winegrowers!*” (“La filière viticole se sent attaquée, le milieu médical se dit sceptique,” *Le Journal de Saône-et-Loire*, 2004)

“*Fetal alcohol syndrome (FAS) is exceptionally rare (0.1 to 0.3% of births)*” (“Une efficacité douteuse,” *Sud Ouest*, 2006)

##### Skirting the alcohol problem (5 occurrences)

The AI claims there are other more important problems than alcoholism during pregnancy and therefore regrets that the pictogram draws all attention onto this one specific issue. It thus proposes setting up pictograms for other (health) problems (3 occurrences, only in period 1):

“*If I have to add this pictogram, I think I will also add ‘forbidden for diabetics', ‘forbidden for people under 16' and ‘forbidden for idiots'*.” (“Vins: la femme enceinte a bon dos…,” *Le Progrès – Lyon*, 2006)

The pictogram is also argued as questionable on the grounds that all human activities are dangerous (1 occurrence, in period 2) and so other more serious issues should be addressed first (1 occurrence, in period 1),:

“*Just as I'd let you have fun coming up with all the pictograms that could be put everywhere in our environment to remind us that the most banal human activities—breathing, eating, driving, sports—all carry risks and that, ultimately, living is bad for your health.”* (“Attention, vivre est nuisible à votre santé,” *Le Bien Public*, 2018)

“*Wine kills fewer people than pharmaceutical drugs, but it is not politically correct to say so*” (“Pas d'eau dans le vin de vignerons sancerrois,” *La Nouvelle République du Centre-Ouest*, 2006)

##### Other arguments (2 occurrences)

Two other more marginally-used arguments claimed that the measure was ridiculous (1 occurrence, during period 1) and unpopular (1 occurrence, during period 1):

“*It's a bit ridiculous and very Franco-French*” (“Le message pour les femmes enceintes n'inquiète pas la filière,” *Le Journal de Saône-et-Loire*, 2007)

“*The issue is unpopular*” (“Zéro alcool pendant la grossesse,” *Le Parisien*, 2004)

#### The warning would have counterproductive effects

##### On the economy (66 occurrences)

The AI argues that this measure attacks the wine sector (43 occurrences, of which 26 in period 2) and weakens producers (12 occurrences, distributed over the two periods):

“*these 64 wine-growing estates denounce ‘the transformation of a product that vectors excellence and is sold across the globe into some kind of contraband […]'*” (“Le logo qui irrite des viticulteurs,” *Midi Libre*, 2018)

“*The labeling of bottles [with the pictogram] also appears to be the last straw for a wine industry already in crisis.”* (“Grossesse sans alcool: les femmes seront prévenues,” *Le Progrès - Lyon*, 2004)

To a lesser extent, the AI raises the point that the producers need time to implement a warning label (3 occurrences, only in period 1), that the cost will be high (3 occurrences, of which 2 in period 2) and that containers carrying the pictogram will be harder to export (2 occurrences, distributed over both periods):

“*For the president of Brasseurs de France, ‘implementing the measure will necessarily take some time, given the time needed to print new labels for our 400 different product references'*.” (“Femmes enceintes: les fabricants d'alcool résignés à apposer un pictogramme,” *AFP Infos Françaises*, 2006)

“*Adding labels or creating back-labels increases our costs”* (“Discrétion assure,” Sud Ouest, 2008)

“*How can we grow exports if wine is considered a dangerous product in France?”* (“Alcool et femmes enceintes: le nouveau logo sur les bouteilles de vin fait polémique chez les vignerons,” *AFP*, 2018)

The measure is seen as binding (1 occurrence, in period 2) and unfair (1 occurrence, in period 1) for producers. They fear that the measure will cause a drop in sales due to lower consumption (1 occurrence, in period 1):

“*new constraints in terms of labelling*” (“Le SAF à l'ordre du jour,” *Emballages magazine* website, 2018)

“*If we have to put it somewhere on the bottle, then all European countries should do it too*.” (“Qu'ils soient plus proches de la réalité,” *L'Union France*, 2017)

##### On women (4 occurrences)

The AI claims that displaying a pictogram that targets pregnant women stigmatizes women and causes guilt (3 occurrences, only in period 1) and anxiety (1 occurrence, in period 2):

“*But what about the risk of guilt-tripping women by labeling the risk*?” (“Alcool: Douste avertit les femmes enceintes,” *Libération*, 2004)

“*It is true that the alcohol lobby considers that informing women would be 'anxiety-provoking'*” (“Alcool: l'inquiétante démission du gouvernement,” *Le Monde*, 2019)

#### Better alternatives exist

##### Promoting responsible consumption and its benefits for health (19 occurrences)

The AI claims that responsible and moderate alcohol consumption is not dangerous, even for pregnant women (10 occurrences, of which *9* in period 1), and that people should drink responsibly (6 occurrences, only in period 1):

“*Alcohol misuse is not recommended for pregnant women, but an occasional drink is not forbidden*” (“Une table ronde pour goûter si le vin est bon,” *Le Progrès – Lyon*, 2005)

“*We should each make sure we are careful, rather than continue calling for new regulations*” (“Petits commerçants,” *La Nouvelle République du Centre-Ouest*, 2004)

They highlight the “benefits” of alcohol consumption (3 occurrences, only in period 1) that get forgotten with this prohibitive warning:

“*André Dubosc [in charge of development at Producteurs Plaimont, a grouping of several wine cooperative structures] also deplores the fact that only the harmful effects of alcohol are taken into account.'I cannot accept that when studies show positive effects, they are not listened to'*.” (“Est-ce la bonne manière d'informer des dangers,” *Sud Ouest*, 2006)

##### Promoting preventive programs (14 occurrences)

The AI proposes launching wide-reaching education and prevention programs aimed at pregnant women, rather than just a simple warning (9 occurrences, of which 5 in period 1):

“*We [the major alcoholic beverage companies in the SAPRO ‘Entreprise et Prévention'] advocate targeted prevention, which is always more complex and often more expensive but ultimately more effective than simple regulatory measures*” (“Douste favorable aux messages sur les bouteilles,” *Le Parisien*, 2004)

Promoting national-scale education on how to responsibly drink wine is another argument put forward (5 occurrences, of which 4 in period 1):

“*Instead of educating people, showing them how to taste [the wine], to enjoy it in moderation, we make them feel guilty. It's shameful*.” (“La filière viticole se sent attaquée, le milieu médical se dit sceptique,” *Le Journal de Saône-et-Loire*, 2004)

##### Promoting the role of health professionals (14 occurrences)

The AI emphasizes that prevention of alcohol exposure in pregnancy is the role of health professionals (and not the producers or legislators through the label) (11 occurrences, of which 10 in period 1):

“*Warning pregnant women is not the legislator's role but the role of doctors*” (“Femmes enceintes et alcool professionnels du vin furieux, les autres partagés,” *AFP*, 2004)

The AI also argues that the warning label is not the right way to display health information as it must not be construed as a medical prescription (3 occurrences, of which 2 in period 1):

“*Are we going to turn our bottles of wine into medical prescriptions?*” (“D'Amécourt et l'esthétique de la femme enceinte,” *Sud Ouest*, 2007)

##### Promoting alcohol-industry collaboration on prevention (14 occurrences)

The AI would like to be part of the policy-making process and criticizes the fact that decisions on the pregnancy warning label (in period 1 and period 2) were taken without consulting with business (6 occurrences, of which 5 in period 2):

“*The councilors denounced what they saw as ‘rushed-through implementation' of a ‘unilaterally imposed' modification brought in ‘without consulting with the wine industry, whereas there were 500,000 vine and wine jobs set to be directly affected by these brutal changes'*” (“Le syndrome d'alcoolisation fœtale en débat,” *Emballages magazine.com*, 2017)

The AI has moved to prove its concern for public health issues. It voiced readiness to engage in prevention initiatives (6 occurrences, of which 4 in period 2) and proposed a “Moderation Council” in period 1 as a public health–private business partnership (2 occurrences):

“*We [members of the interprofessional bureau of Burgundy wines] are absolutely ready to engage in consumer education and awareness action on the dangers of alcohol abuse*.”

(“Etiquetage sur les bouteilles premières réactions,” *Le Bien Public*, 2004)

“…*the creation of a ‘Moderation Council' that deliver targeted communication to pregnant women*” (“Femmes enceintes et alcool: professionnels du vin furieux, les autres partagés,” *AFP*, 2004)

Beyond these arguments against the warning-label measure, there was a fairly marginal minority of AI agents in favor of the measure. Their arguments are described below.

### Arguments in favor of the pregnancy warning label

Two categories of pro-pictogram arguments emerged: (1) positions in favor of implementation (in period 1), (2) positions in favor of an expanded pictogram (in period 2). These categories are outlined and materialized *via* the examples given below.

#### In favor of implementation (12 occurrences)

Some of the actors were unconditionally in favor of implementing the pregnancy warning pictogram (8 occurrences) and sometimes even promised to spread the “abstinence during pregnancy” message even more widely. This pro message was mainly promoted by individual winegrowers. They were ready to implement the pictogram after seeing shocking media coverage of the “Lille affair” in 2004, a lawsuit brought by mothers whose children were victims of FAS. The mothers went to court because of the lack of information about the dangers of alcohol consumption during pregnancy (from doctors and labeling) at the time of the trial (7, 42, 43) (2 occurrences):

“*Woken up by the Lille affair [during which an association filed a complaint for failure to inform pregnant women about the dangers of alcohol use], he did not want to wait for the new legislation. His 600,000 bottles of Château Puech-Haut will now carry a statement that reads ‘not advised for pregnant women' in four languages*.” (“Grossesse sans alcool les femmes seront prévenues,” *Le Progrès – Lyon*, 2004)

One other actor, a SAPRO that represents the AI (“Entreprises et Prévention”), was also in favor of warnings on condition that the effort was a shared one:

“*We are not against labelling bottles with health messages”, says Arnaud Lassince, general manager of Entreprises et Prévention that federates 18 of the largest French alcohol industries and represents 150 brands of alcoholic beverages*. “*We are not lobbying against it we are well aware of the issues. But we don't want to be the only ones who have to get the message through.”* (“Alcool et grossesse: le cri d'alarme des spécialistes,” *Le Parisien*, 2004)

#### In favor of expansion (18 occurrences)

During period 2, some actors (mainly promoted by big alcohol companies grouped in trade associations: Fédération française des spiritieux, Fédération française des vins d'apéritif, Vin & Société, Brasseurs de France, or SAPROs: Avec Modération) were in favor of doubling the size of the pictogram and doubling the prevention effort (7 occurrences) if a compromise was proposed (4 occurrences), if the size did not exceed a certain threshold (cited as “doubled” by one actor and 8 mm maximum by two actors), or if there was grace period before implementation (2 occurrences):

“*The profession proposes that the sticker should double in size, to 0.8 mm instead of 0.3 or 0.4 mm, and be printed with greater contrast. The profession also commits to widely disseminate the ‘no alcohol while pregnant' message”* (“Le lobby de l'alcool va financer la lutte contre l'alcoolisme,” *Sciences et Avenir website*, 2018)

“*If we're proposed a two-centimeter pictogram, that might be a problem, because our labels are small. But we should be able to find a compromise by playing on the colors to make it more visible; the issue is not opposition to the measure*” (“La filière viticole sous pression pour clarifier ses étiquettes,” *Corse-Matin*, 2018)

Some actors were sometimes supportive without specifying conditions (2 occurrences):

“*Deemed too discreet by the health authorities, the expansion of the pictogram prohibiting alcohol for pregnant women is one of the measures proposed [proposed by the three alcohol sectors in their contribution to the national public health plan]*” (“Alcool et dose de santé publique,” *Corse-Matin*, 2018)

## Discussion

A growing public health literature on the influence of companies selling unhealthy products like alcohol that are bad for population health has converged around the emerging concept of commercial determinants of health (25, 44, 45). Lobbying is one of the channels through which companies influence public policy (45). The AI mobilizes lobbying strategies in an effort to counter effective alcohol prevention measures (minimum unit pricing, marketing regulations, etc.) (46, 47), including labeling (6). It is well-known that in the context of tobacco, warnings improve consumer knowledge and influence smoking behaviors (48). However, in the context of alcohol, warnings are an under-developed health measure: few countries have adopted alcohol warnings, and the warnings that have been adopted tend to be poorly designed (7). One reason for this situation is AI lobbying against this measure (13, 23). Lobbying strategies and arguments against alcohol warnings have rarely been analyzed in the literature. Our research fills this gap by analyzing the arguments used by AI lobbyists in the French mainstream press in their defense against alcohol warnings targeted pregnant women.

### Main results of the research

This research reveals that the AI responds to new public health proposals *via* reactions in the mainstream press. Indeed, peaks in number of publications were found to coincide with the warning-label implementation (in and around 2007) and with the pictogram expansion-project schedule (in and around 2018). This shows that the AI uses the mainstream press as an indirect tool for lobbying, as previously identified (20, 38). As the number of articles through the press is higher in 2018 compared to 2007, it may be due to professionalization of strategies of lobbying of the AI (4) that could consist of a wider use of the press to spread arguments and thus indirectly influence opinion leaders and decision makers.

This research also found that the AI tends to develop transnational arguments to counter warnings in general and those that target pregnant women in particular in different countries. Some arguments identified in this French research have also been identified in the extant research on lobbying against alcohol warnings in Anglosphere countries (12, 13, 23), i.e., that the warnings are ineffective in changing behaviors, that warnings would have counterproductive effects on women and the economy, and that there are better alternatives, such as public information campaigns which are actually less effective than population-based interventions. Interestingly, we also surfaced arguments that emerge specifically in France, showing that lobbying seems to be adapted to nation-specific features (36). For instance, some arguments emphasize how wine and winemaking hold a special place in the French culture, with the idea that “wine is not an alcohol like others” (49), and a special position in the French economy (36). As wine is deeply embedded in French culture (50) and given the strong proximity between the French President and this wine sector (details to come), the AI may have adapted its strategies and uses the voice of winemakers in the press as a kind of credible and appreciated front group.

Our research has also emerged new arguments not previously captured in research on lobbying against warnings. We identified arguments in favor of the measure (even though they represent a tiny minority compared to those against), some of which were voiced by independent winemakers in period 1. They were ready to implement the pictogram after seeing shocking media coverage of a lawsuit brought by three mothers whose children were victims of FAS on the grounds that they had been under informed on the dangers of alcohol consumption during pregnancy (7, 42). During period 2, the AI also agreed to display a pictogram with a minimum size of 8 mm and enhanced by color contrast (51). Contrary to period 1, this discourse in favor of the warnings mainly came from organizations like “Vin et Société,” funded by the AI and identified as a strong front-group lobbyist in France (4, 36). Those pro-measure arguments may be part of a process of professionalized lobbying by the AI that includes the recent rise of corporate social responsibility (CSR) strategies (4, 52). CSR strategies include launching prevention campaigns (52), a willingness to collaborate with governments to secure solutions (i.e., the Responsibility Deal in the UK) (53), engagement with safer drinking by offering low and no-alcohol products (4), and proposed self-regulated marketing in order to protect young and vulnerable people (36, 46). Considering this CSR trend that is an indirect lobbying tactic used to improve image of companies (46), the arguments that emerged in France in favor of pregnancy warning labels may stem from this trend developed by the AI worldwide, in a similar way to those employed by the tobacco industry (54, 55).

Our research highlights that whereas arguments used during the two periods were fairly similar in terms of content and number, the AI's lobbying failed in period 1 (warnings were adopted) and won in period 2 (the expansion project was aborted). It is difficult to interpret this finding, but part of the explanation may be different political and social contexts (36, 56). Period 1 was marked by high media coverage of a lawsuit brought by three mothers (the “Lille affair”) whose children were victims of FAS (7, 42). This media coverage may have put added pressure on government to implement the alcohol warnings. Period 2 was characterized by an extremely favorable political context, especially for the wine industry. It is well-known that the French President Emmanuel Macron has very close ties with the AI (32) and regularly expresses himself publicly as a defender of wine, which he qualified as “inseparable from our art of living, this art of being French” (57, 58) and he claimed to journalists that he drinks wine “daily, lunch and dinner” (58, 59). For his engagement in favor of wine, he was elected “Personality of the Year 2022” by the Revue du Vin de France (“French Wine Review”), he accepted this prize and received it in person (33, 34). From 2017 to 2019, he also hired Audrey Bourolleau, the former manager of the SAPRO “Vin et Société,” that joined the French government as an agricultural adviser (36). Regarding alcohol prevention, Emmanuel Macron publicly disavowed the former Health Minister Agnès Buzyn who wanted to increase the size of the pregnant warning in 2018 (60). These different examples may explain the success of the AI in the period 2 in blocking the enlargement of the pictogram.

### Limitations of the research

This research has some limitations. First, it has taken into account the position of the AI in order to identify the arguments it uses. It would be instructive for future research to analyze the arguments relayed by actors in health, both to determine their weight in the mainstream press (are they under/over-represented compared to the AI?) and to analyze the pro-health arguments used (content of the arguments and their pattern of change over time). Second, this analysis was based on the mainstream press. Other potentially-relevant media should be analyzed, such as the trade press, parliamentary documents, or social media (39, 61, 62) in order to gain external validity and identify arguments from other sources. Finally, the study may be exposed to the biases inherent to the use of a qualitative methodology, such as interpretation bias. We tried to limit this bias by working among the team to find a consensus when doubts emerged on some press articles.

### Theoretical contributions

This research makes two main contributions to the scientific literature. First, it provides a larger framework for analyzing the arguments of the AI lobbying against alcohol warnings in general and those aimed at pregnant women in particular. Second, the analysis led here was conducted over a long period of time (which is rare in the literature) on a health measure proposed by actors in health that has been adopted or not in a non-Anglosphere country (13, 20) where the context is a priori favorable to alcohol (32, 36).

### Public health contributions and recommendations

Based on our findings, we make five public health recommendations to tackle the issue of AI lobbying against health warnings (and against public health measures in general).

First, given the strength (and effectiveness) of lobbying at national level, international treaties are needed to counter national-level influence on individual governments. A European Union Directive on alcohol warnings would be a relevant option, as already done for tobacco in 2014 requiring a combined health warning consisting of a picture, a text warning, and information on stopping smoking, covering 65% of the front and back of cigarette packs (63). Beyond the European Union level, a “Framework Convention on Alcohol Control” would also be relevant, as already done for tobacco in 2005 (the FCTC: Framework Convention on Tobacco Control) (64) wherein article 11 stipulates that each Party signing and ratifying the treaty is to adopt and implement effective labeling measures within a period of three years. For countries similar to France very close to the AI, the article 5.3 of the FCTC that stipulates that each Party has to protect its “policies from commercial and other vested interests of the tobacco industry in accordance with national law” (64) should be replicated to the AI in order to protect alcohol policies.

Second, the French example appears to highlight strong links between the AI and government (32, 36, 57) that could explain why certain public health measures do not get adopted. To counter this problem, citizens and health actors need to be better informed on these links *via* transparency instruments that could limit interference in public decisions. National legislation could be proposed on alcohol, following the example of the French law on transparency of the tobacco industry's influence relations, in particular on expenses related to influencing or representing the interests of tobacco product manufacturers, importers and distributors and their representatives (Article 26 of January 26, 2016) (65). This could go further by compelling the AI to disclose any and all expenses tied to indirect lobbying channels (research funding, presence and participation of the AI in public commissions) (66).

Third, more research is needed on the arguments used by the AI against alcohol warnings. To counter the AI's argument challenging the effectiveness of pregnancy warning labels, more studies should be conducted on the content and design of effective warnings. There is limited research on alcohol warnings compared to tobacco warnings at international level and especially in France where only three studies (10, 15, 16) have been published on these issues. Concerning the argument around the economic costs for the AI, more research is needed on the economic burden of alcohol for society. In France, the first (and only) research that estimated this social cost dates from 2010 and arrived at a figure of 120 billion euros per year (67). No research has been carried out since to update this figure.

Fourth, it is vital to provide actors in health (NGOs, public institutes, health professionals) with more training and skills in order to make them more effective in lobbying tactics and press relations and adopt similar strategies of the AI to better counter them.

Finally, given that denouncing the industry's marketing and lobbying tactics seems to be effective, counter-marketing campaigns should be implemented, as was done in tobacco with the “Truth” campaign in the USA (68). It could be useful to develop a campaign *via* social media to denounce AI lobbying. The effectiveness of counter-marketing campaigns is explained by inoculation theory, which posits that people can be protected from attempts at commercial manipulation if they are warned against them with counter-arguments (69, 70).

## Author's note

Our research fills a gap in a limited and mainly Anglosphere research on lobbying against alcohol warnings. We conducted an analysis of the arguments advanced by the alcohol industry in the French mainstream press over a 20-year period covering a failure of lobbying (introduction of the measure in 2007) and a success of lobbying (failure of the pictogram expansion project in 2018). Our research found some arguments that were similar to those already identified in the literature, but it also surfaced other arguments that have emerged, probably due to the specificity of France as a pro-wine country and probably also linked to the emergence of CSR strategies. An analytical framework of arguments used by the alcohol industry is suggested, which could be helpful for countries that have a long history of pro-alcohol culture. Various recommendations are suggested in order to counter these alcohol industry's arguments: (1) implementation of international treaties in order to counter national-level influence on governments; (2) implementation of national legislation for more transparency around the alcohol industry's influence; (3) development of research on health measures against alcohol use; (4) educating actors in health on lobbying; (5) implementation of counter-marketing campaigns to expose and delegitimize the alcohol industry's lobbying practices.

## Data availability statement

The original contributions presented in the study are included in the article/Supplementary material, further inquiries can be directed to the corresponding author/s.

## Author contributions

AM organized the database, performed the analysis, and wrote the first draft of the manuscript. MS organized the database and performed the analysis. KG-M conceived the study. All authors contributed to manuscript revision and read and approved the final submitted version.

## Funding

AM was partially funded by Addictions France.

## Conflict of interest

The authors declare that the research was conducted in the absence of any commercial or financial relationships that could be construed as a potential conflict of interest.

## Publisher's note

All claims expressed in this article are solely those of the authors and do not necessarily represent those of their affiliated organizations, or those of the publisher, the editors and the reviewers. Any product that may be evaluated in this article, or claim that may be made by its manufacturer, is not guaranteed or endorsed by the publisher.
