# Supplementary material for: How the alcohol industry fought against pregnancy warning labels in France. A press coverage analysis spanning 20 years
Source: Front Public Health. 2022 Aug 26;10:933164. doi: 10.3389/fpubh.2022.933164 (PMC9458884; doi:10.3389/fpubh.2022.933164)
Supplement: Supplementary file 1 [file Data_Sheet_1.PDF]

SUPPLEMENTARY MATERIAL

Appendix 1. French mandatory alcohol warning labels aimed at pregnant women

<table>
<tr><th></th><th colspan="2">A text message OR a pictogram</th></tr>
<tr><td>Current Acts</td><td colspan="2"> Article L3322-2 du Code de la santé publique : «Toutes les unités de conditionnement des boissons alcoolisées portent, dans les conditions fixées par arrêté du ministre chargé de la santé, un message à caractère sanitaire préconisant l'absence de consommation d'alcool par les femmes enceintes.» (“All packaging units of alcoholic beverages shall display, under the conditions laid down by order of the Minister for Health, a health message advising pregnant women not to drink alcohol.”) Arrêté du 2 octobre 2006 relatif aux modalités d’inscription du message à caractère sanitaire préconisant l’absence de consommation d’alcool par les femmes enceintes sur les unités de conditionnement des boissons alcoolisées : «Le message sanitaire [...] figure dans le même champ visuel que l'indication obligatoire relative au titre alcoométrique volumique.» «Le message sanitaire [...] est inscrit sur fond contrastant, de manière à être visible, lisible, clairement compréhensible, indélébile. Il ne doit en aucune façon être dissimulé, voilé ou séparé par d'autres indications ou images.» (“The health message [...] shall appear in the same visual field as the mandatory indication of alcoholic strength by volume.” “The health message [...] shall be printed on a contrasting background on a way that makes it visible, readable, clearly understandable and indelible. It must not be concealed, obscured or separated by other indications or images in any way.”) </td></tr>
<tr><td>Content</td><td> « La consommation de boissons alcoolisées pendant la grossesse, même en faible quantité, peut avoir des conséquences graves sur la santé de l’enfant » (“Consumption of alcoholic beverages during pregnancy, even in small quantities, can have serious consequences for the health of the child”) </td><td>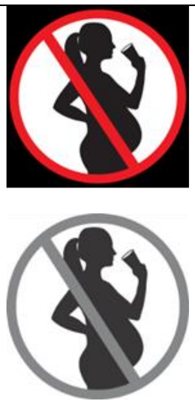
Two possible versions of the pictogram (color-contrasted or black-and-white versions)</td></tr>
</table>

<table>
<tr>
<td>Examples</td>
<td>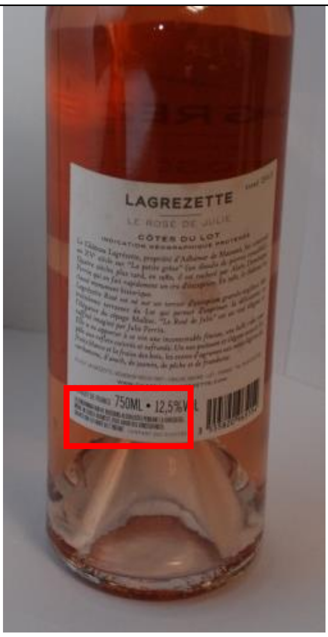

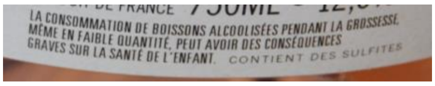
</td>
<td>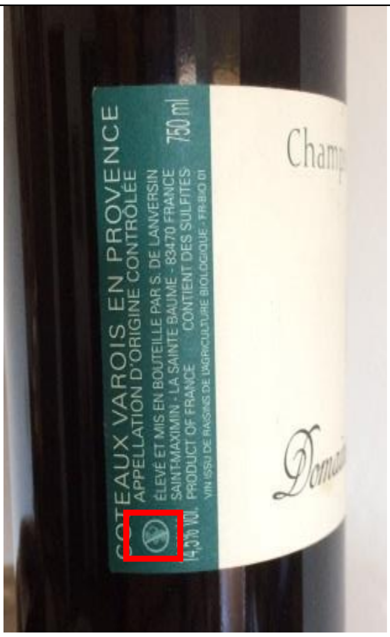
</td>
</tr>
</table>

APPENDIX 2. References of the 85 press articles included

Author	Title	Journal	Url	Date
Stéphane Horel et Pascale Santi	Alcool : comment le lobby sape la prévention	Le Monde	https://nouveau.europresse.com/Link/U032196T_1/news%c2%b720200122%c2%b7LM%c2%b7659398	22/01/2020
Pascale Santi et Stéphane Horel	Comment le lobby de l'alcool sape toute prévention prônant l'abstinence	Le Monde (site web)	https://nouveau.europresse.com/Link/U032196T_1/news%c2%b720200120%c2%b7LMF%c2%b76026616_1650684	20/01/2020
	D'Amécourt et l'esthétique de la femme enceinte La mise en place, ces derniers jours, de la nouvelle campagne de prévention visant la consommation d'alcool pour les femmes enceintes n'est pas du goût de l'élu de Sauveterre-de-Guyenne.	Sud Ouest	https://nouveau.europresse.com/Link/U032196T_1/news%c2%b720071009%c2%b7SO%c2%b7091007ap962777	09/10/2007
A. Spicher	Vins : la femme enceinte a bon dos...	Le Progrès - Lyon	https://nouveau.europresse.com/Link/U032196T_1/news%c2%b720061125%c2%b7PR%c2%b70200611 25_jura_chassieu_090	25/11/2006
Annie GAUCHER	Pas d'eau dans le vin des vignerons sancerrois	La Nouvelle République du Centre-Ouest	https://nouveau.europresse.com/Link/U032196T_1/news%c2%b720060605%c2%b7NR%c2%b7701114777	05/06/2006
César Compadre	« Une efficacité douteuse »	Sud Ouest	https://nouveau.europresse.com/Link/U032196T_1/news%c2%b720061017%c2%b7SO%c2%b71710 06a44598	17/10/2006
César Compadre	Discrétion assurée	Sud Ouest	https://nouveau.europresse.com/Link/U032196T_1/news%c2%b720080304%c2%b7SO%c2%b70403 08ap2006664	04/03/2008

MARIE-JOSEE COUGARD	Naissance difficile de l'étiquetage préventif des boissons alcoolisées	Les Echos	https://nouveau.europresse.com/Link/U032196T_1/news%c2%b720060926%c2%b7EC%c2%b74475429	26/09/2006
G. B.	« Est-ce la bonne manière d'informer des dangers ? »	Sud Ouest	https://nouveau.europresse.com/Link/U032196T_1/news%c2%b720060513%c2%b7SO%c2%b7130506a106271	13/05/2006
Cécile Prieur	M. Douste-Blazy souhaite un étiquetage des bouteilles d'alcool	Le Monde	https://nouveau.europresse.com/Link/U032196T_1/news%c2%b720040807%c2%b7LM%c2%b70q0708_890804	07/08/2004
Jean VALBAY	Alcool et femmes enceintes : enquête judiciaire pour défaut d'information	Le Figaro	https://nouveau.europresse.com/Link/U032196T_1/news%c2%b720040805%c2%b7LF%c2%b720040805%c3%972fig0170	05/08/2004
	Pression sur l'étiquette	Sud Ouest	https://nouveau.europresse.com/Link/U032196T_1/news%c2%b720090423%c2%b7SO%c2%b7230409ap4440552	23/04/2009
	Le message pour les femmes enceintes n'inquiète pas la filière	Le Journal de Saône et Loire	https://nouveau.europresse.com/Link/U032196T_1/news%c2%b720071010%c2%b7SA%c2%b720071010%c3%972%c3%971jsa0287	10/10/2007
	Femmes enceintes: message sanitaire ou logo sur les bouteilles d'alcool (JO)	AFP Economiques	https://nouveau.europresse.com/Link/U032196T_1/news%c2%b720061003%c2%b7FE%c2%b7135658-tx-tkd06	03/10/2006
	La filière viticole se sent attaquée, le milieu médical se dit sceptique	Le Journal de Saône et Loire	https://nouveau.europresse.com/Link/U032196T_1/news%c2%b720040809%c2%b7SA%c2%b720040809%c3%972%c3%971jsa0251	09/08/2004
Annie GAUCHER	Réunion d'après-vendanges aux caves de la Mignonne	La Nouvelle République du Centre-Ouest	https://nouveau.europresse.com/Link/U032196T_1/news%c2%b720051109%c2%b7NR%c2%b705239120	09/11/2005

	La vente de floc est à la baisse	Sud Ouest	https://nouveau.europresse.com/Link/U032196T_1/news%c2%b720070326%c2%b7SO%c2%b7260307a4930	26/03/2007
Par Christian CHARCOSSEY	Femmes enceintes: les fabricants d'alcool résignés à apposer un pictogramme (PAPIER D'ANGLE)	AFP Infos Françaises	https://nouveau.europresse.com/Link/U032196T_1/news%c2%b720060512%c2%b7AF%c2%b7090007-tx-rbo21	12/05/2006
Julien HAMELIN.	L'alcool nuit gravement à la santé du foetus	Ouest-France	https://nouveau.europresse.com/Link/U032196T_1/news%c2%b720040805%c2%b7OF%c2%b743771009	05/08/2004
ECOIFFIER Matthieu	Alcool : Douste avertit les femmes enceintes	Libération	https://nouveau.europresse.com/Link/U032196T_1/news%c2%b720041020%c2%b7LI%c2%b70li20041020103	20/10/2004
LAMOUREUX Marine	Santé publique. Les méfaits de l'alcool sur le foetus sont sous-estimés. Le ministre de la santé s'est dit hier favorable à ce qu'une information sur ces dangers figure sur l'étiquette des bouteilles.	La Croix	https://nouveau.europresse.com/Link/U032196T_1/news%c2%b720040806%c2%b7LC%c2%b70040806lc_inx035	06/08/2004
C. B.	Une étiquette comme aux Etats-Unis ?	Le Progrès - Lyon	https://nouveau.europresse.com/Link/U032196T_1/news%c2%b720040805%c2%b7PR%c2%b70200408 05_ig_013	05/08/2004
Catherine PETITNICOLAS	Les sénateurs ne veulent pas protéger le foetus contre l'alcool	Le Figaro	https://nouveau.europresse.com/Link/U032196T_1/news%c2%b720040214%c2%b7LF%c2%b720040214%c3%972fig0140	14/02/2004
Christine Beranger	Grossesse sans alcool les femmes seront prévenues	Le Progrès - Lyon	https://nouveau.europresse.com/Link/U032196T_1/news%c2%b720041007%c2%b7PR%c2%b70200410 07_ig_033	07/10/2004

	Etiquetage sur les bouteilles : Premières réactions	Le Bien Public	https://nouveau.europresse.com/Link/U032196T_1/news%c2%b720040806%c2%b7BP%c2%b720040806%c3%972%c3%971bpa0005	06/08/2004
	Alcool et grossesse : un nouveau-né touché chaque jour	Le Progrès (Lyon)	https://nouveau.europresse.com/Link/U032196T_1/news%c2%b720190629%c2%b7PR%c2%b741599555416	29/06/2019
	Alcool : l'inquiétante démission du gouvernement	Le Monde	https://nouveau.europresse.com/Link/U032196T_1/news%c2%b720190123%c2%b7LM%c2%b734365	23/01/2019
	Alcool et grossesse : des conséquences sur un nouveau-né chaque jour	Le Progrès - Lyon	https://nouveau.europresse.com/Link/U032196T_1/news%c2%b720180917%c2%b7PR%c2%b7402823852047	17/09/2018
	Trop de bébés trinquent	Le Courrier de l'Ouest	https://nouveau.europresse.com/Link/U032196T_1/news%c2%b720180912%c2%b7OCO%c2%b7mjaxoc0woty3zty0mwnlowywzwnjmjhkotg4ntuyywfizjy4oda1	12/09/2018
	Le SAF à l'ordre du jour	Emballages magazine (site web)	https://nouveau.europresse.com/Link/U032196T_1/news%c2%b720180906%c2%b7GEMB%c2%b746876	06/09/2018
	Les dangers de l'alcool pendant la grossesse	Courrier picard	https://nouveau.europresse.com/Link/U032196T_1/news%c2%b720180906%c2%b7VNC%c2%b720180690688	06/09/2018
Camille Gaubert	L'alcoolisation foetale concerne une naissance par jour en France	Sciences et Avenir (site web)	https://nouveau.europresse.com/Link/U032196T_1/news%c2%b720180904%c2%b7SAW%c2%b7127199	04/09/2018
Avec Afp	Alcoolisation foetale: les Hauts-de-France très touchés	La Voix du Nord (site web)	https://nouveau.europresse.com/Link/U032196T_1/news%c2%b720180904%c2%b7VWN%c2%b7019	04/09/2018

	Les élus s'opposent au nouveau logo alcool	L'Union (France)	https://nouveau.europresse.com/Link/U032196T_1/news%c2%b720180718%c2%b7VNU%c2%b720180718243	18/07/2018
Frank Mauerhan	Attention, vivre est nuisible à votre santé	Le Bien Public	https://nouveau.europresse.com/Link/U032196T_1/news%c2%b720180718%c2%b7BP%c2%b7402410644363	18/07/2018
	Le logo qui irrite des viticulteurs	Midi Libre	https://nouveau.europresse.com/Link/U032196T_1/news%c2%b720180714%c2%b7ML%c2%b739934945	14/07/2018
	Alcool et femmes enceintes : la taille du logo fait polémique chez les vignerons	L'Indépendant	https://nouveau.europresse.com/Link/U032196T_1/news%c2%b720180714%c2%b7MN%c2%b739937366	14/07/2018
	Femmes enceintes : le logo qui fâche	La Dépêche du Midi	https://nouveau.europresse.com/Link/U032196T_1/news%c2%b720180714%c2%b7DPM%c2%b739931972	14/07/2018
	Alcool et femmes enceintes : polémique des vignerons sur le logo	Le Petit Bleu d'Agen	https://nouveau.europresse.com/Link/U032196T_1/news%c2%b720180714%c2%b7DPP%c2%b739937232	14/07/2018
	Alcool et femmes enceintes: polémique des vignerons sur le logo	Centre Presse Aveyron	https://nouveau.europresse.com/Link/U032196T_1/news%c2%b720180714%c2%b7MF%c2%b739937437	14/07/2018
genst01	Le logo déconseillant l'alcool aux femmes enceintes " mortifère "	La Nouvelle République du Centre-Ouest	https://nouveau.europresse.com/Link/U032196T_1/news%c2%b720180714%c2%b7NR%c2%b71111644583	14/07/2018
AFP	Alcool et femmes enceintes : le nouveau logo sur les bouteilles de vin fait polémique chez les vignerons	L'Indépendant (site web)	https://nouveau.europresse.com/Link/U032196T_1/news%c2%b720180713%c2%b7MNE%c2%b7199	13/07/2018

Eric Hacquemand	Vin : Agnès Buzyn voit rouge	Paris Match (site web)	https://nouveau.europresse.com/Link/U032196T_1/news%c2%b720180712%c2%b7LPB%c2%b7008	12/07/2018
	Agnès Buzyn s'emballe autour d'un logo anti-alcool	Le Monde.fr	https://nouveau.europresse.com/Link/U032196T_1/news%c2%b720180628%c2%b7LMF%c2%b75322693	28/06/2018
MARIE-JOSEE COUGARD	Alcoolisme : les professionnels prêts à financer la prévention	Les Echos	https://nouveau.europresse.com/Link/U032196T_1/news%c2%b720180628%c2%b7EC%c2%b70301888440505	28/06/2018
	Un nouveau dispositif contre le SAF	Emballages magazine.com	https://nouveau.europresse.com/Link/U032196T_1/news%c2%b720180627%c2%b7GEMB%c2%b746288	27/06/2018
Le Parisien avec AFP	En France, c'est le lobby de l'alcool qui va financer la lutte anti-alcoolisme	Aujourd'hui en France (site web)	https://nouveau.europresse.com/Link/U032196T_1/news%c2%b720180627%c2%b7PJW%c2%b777796805	27/06/2018
Eric Favereau	Alcoolisme féminin : un fléau sans modération	Libération	https://nouveau.europresse.com/Link/U032196T_1/news%c2%b720180509%c2%b7LI%c2%b730f80de6763edf5c6960c376aab04ac7	09/05/2018
	Alcool et santé : c'est la bagarre	Le Courrier de l'Ouest	https://nouveau.europresse.com/Link/U032196T_1/news%c2%b720180505%c2%b7OCO%c2%b75793794	05/05/2018
	Le SAF fait son retour	Emballages magazine.com	https://nouveau.europresse.com/Link/U032196T_1/news%c2%b720180418%c2%b7GEMB%c2%b745430	18/04/2018
ccolinet	Centimètres	Centre Presse (Vienne)	https://nouveau.europresse.com/Link/U032196T_1/news%c2%b720180418%c2%b7NCP%c2%b71111340912	18/04/2018

	Le pictogramme pour femmes enceintes arrive cet été	NouvelObs.com	https://nouveau.europresse.com/Link/U032196T_1/news%c2%b720060512%c2%b7OA%c2%b720060512%c3%972obs7421	12/05/2006
	Une table ronde pour goûter si le vin est bon	Le Progrès – Lyon	https://nouveau.europresse.com/Link/U032196T_1/news%c2%b720050822%c2%b7PR%c2%b7020050822_jura_chassieu_090	22/08/2005
Simone Tomasin	Des viticultrices à l'Assemblée	Sud Ouest	https://nouveau.europresse.com/Link/U032196T_1/news%c2%b720100208%c2%b7SO%c2%b7080210ap5688582	08/02/2010
Florence Deguen	Alcool et grossesse : le cri d'alarme des spécialistes	Le Parisien	https://nouveau.europresse.com/Link/U032196T_1/news%c2%b720040805%c2%b7PA%c2%b7241099990	05/08/2004
Philippe ZYGEL	"Déconseillé aux femmes enceintes" : un vigneron affiche la couleur (MAGAZINE)	AFP Infos Economiques	https://nouveau.europresse.com/Link/U032196T_1/news%c2%b720041002%c2%b7FE%c2%b7105028-tx-ori82	02/10/2004
M. Bruno BOUGES, de Mosnes (Indre-et-Loire).	Petits commerçants	La Nouvelle République du Centre-Ouest	https://nouveau.europresse.com/Link/U032196T_1/news%c2%b720040907%c2%b7NR%c2%b7013481400	07/09/2004
	Qu'ils soient plus proches de la réalité	L'Union (France)	https://nouveau.europresse.com/Link/U032196T_1/news%c2%b720170418%c2%b7VNU%c2%b720170418385	18/04/2017
	Zéro alcool durant la grossesse !	Le Progrès de Fécamp	https://nouveau.europresse.com/Link/U032196T_1/news%c2%b720170405%c2%b7PAN%c2%b777875147	05/04/2017
	Le syndrome d'alcoolisation foetale en débat	Emballages magazine.com	https://nouveau.europresse.com/Link/U032196T_1/news%c2%b720170313%c2%b7GEMB%c2%b740137	13/03/2017

Le Figaro Vin	La filière viticole vent debout contre un projet d'étiquetage agrandi pour femmes enceintes	Le Figaro.fr	https://nouveau.europresse.com/Link/U032196T_1/news%c2%b720170121%c2%b7LFF%c2%b7129114	21/01/2017
	Non au logo agrandi pour femmes enceintes	L'Union (France)	https://nouveau.europresse.com/Link/U032196T_1/news%c2%b720170121%c2%b7VNU%c2%b720170121312	21/01/2017
	Projet contesté	Le Télégramme (Bretagne)	https://nouveau.europresse.com/Link/U032196T_1/news%c2%b720170121%c2%b7TL%c2%b7100778050	21/01/2017
	Des viticulteurs bordelais entrent en guerre contre le logo "femme enceinte"	L'Express (site web)	https://nouveau.europresse.com/Link/U032196T_1/news%c2%b720170120%c2%b7EWL%c2%b7xpr1871236	20/01/2017
	Les viticulteurs bordelais en guerre contre le logo agrandi pour femmes enceintes	Sud Ouest (site web)	https://nouveau.europresse.com/Link/U032196T_1/news%c2%b720170120%c2%b7SOE%c2%b7442	20/01/2017
	Le logo qui enfante les polémiques	Sud Ouest	https://nouveau.europresse.com/Link/U032196T_1/news%c2%b720170119%c2%b7SO%c2%b7190117ap5897870	19/01/2017
	Désaccords autour d'un logo	Emballages magazine.com	https://nouveau.europresse.com/Link/U032196T_1/news%c2%b720170117%c2%b7GEMB%c2%b739258	17/01/2017
C.S.	Réglementation : Les pouvoirs publics veulent agrandir le pictogramme femme enceinte	Rayon Boissons (site web)	https://nouveau.europresse.com/Link/U032196T_1/news%c2%b720170117%c2%b7BKZ%c2%b7003	17/01/2017
	Douste favorable aux messages sur les bouteilles	Le Parisien	https://nouveau.europresse.com/Link/U032196T_1/news%c2%b720040806%c2%b7PA%c2%b72411000393	06/08/2004

Béatrice Peyrani	« Je crains que l'ISF ne me pousse à quitter la France »	Le Point	https://nouveau.europresse.com/Link/U032196T_1/news%c2%b720060629%c2%b7PO%c2%b7176307401	29/06/2006
	Alcool : « Entreprise et prévention » lutte contre les abus	Le Progrès - Lyon	https://nouveau.europresse.com/Link/U032196T_1/news%c2%b720070219%c2%b7PR%c2%b7020070219_jura_chassieu_107	19/02/2007
Christian CHARCOSSEY	Femmes enceintes et alcool: professionnels du vin furieux, les autres partagés	AFP Infos Economiques	https://nouveau.europresse.com/Link/U032196T_1/news%c2%b720040805%c2%b7FE%c2%b70ecf565_218_200559	05/08/2004
DOMINIQUE MYRIAM DORNIER	Deux soeurs, du vin et des livres	La Tribune - Acteurs de l'économie - Rhône-Alpes	https://nouveau.europresse.com/Link/U032196T_1/news%c2%b720141201%c2%b7TRA%c2%b70052	01/12/2014
	Alcoolisme: s'attaquer à "l'abus d'alcool" et non "à son usage" (producteurs)	AFP Infos Economiques	https://nouveau.europresse.com/Link/U032196T_1/news%c2%b720070209%c2%b7FE%c2%b7154924-tx-qsx38	09/02/2007
Sciences et Avenir avec AFP	Absence de financement pour lutter contre l'addiction à l'alcool	Sciences et Avenir (site web)	https://nouveau.europresse.com/Link/U032196T_1/news%c2%b720181012%c2%b7SAW%c2%b7128479	12/10/2018
	[Alcool et dose de santé publique...]	Corse-Matin	https://nouveau.europresse.com/Link/U032196T_1/news%c2%b720180703%c2%b7NIC%c2%b717855396	03/07/2018
	Alcoolisme: Le plan de prévention du lobby de l'alcool peut-il être efficace?	20 Minutes (site web)	https://nouveau.europresse.com/Link/U032196T_1/news%c2%b720180628%c2%b7VIW%c2%b7139	28/06/2018
	Front uni contre l'alcoolisme	Presse Océan	https://nouveau.europresse.com/Link/U032196T_1/news%c2%b720180704%c2%b7OFP%c2%b7mjax	04/07/2018

			oc0wn2iyyzm2mmziytzhntu0y2y0zdq2mja5zmrjmjkwmmm3	
	La filière viticole sous pression pour clarifier ses étiquettes	Corse-Matin	https://nouveau.europresse.com/Link/U032196T_1/news%c2%b720180418%c2%b7NIC%c2%b717303665	18/04/2018
Sciences et Avenir avec AFP	Le lobby de l'alcool va financer la lutte contre l'alcoolisme	Sciences et Avenir (site web)	https://nouveau.europresse.com/Link/U032196T_1/news%c2%b720180627%c2%b7SAW%c2%b7125356	27/06/2018
	Les principales mesures	Charente Libre	https://nouveau.europresse.com/Link/U032196T_1/news%c2%b720180628%c2%b7CHA%c2%b78277039	28/06/2018
Le Figaro.fr avec AFP	Les producteurs d'alcool se lancent dans la prévention	Le Figaro.fr	https://nouveau.europresse.com/Link/U032196T_1/news%c2%b720180627%c2%b7LFF%c2%b720180627filwww00079	27/06/2018
ÉRIC BOSCHMAN	On a marché sur la tête!	Le Soir	https://nouveau.europresse.com/Link/U032196T_1/news%c2%b720130928%c2%b7SR%c2%b731wa4q	28/09/2013
	Un logo femme enceinte sur les étiquettes	Le Bien Public	https://nouveau.europresse.com/Link/U032196T_1/news%c2%b720071004%c2%b7BP%c2%b720071004%c3%972%c3%971bpa0091	04/10/2007
Marc Payet	Zéro alcool pendant la grossesse	Le Parisien	https://nouveau.europresse.com/Link/U032196T_1/news%c2%b720041022%c2%b7PA%c2%b7241172822	22/10/2004

APPENDIX 3. Alcohol-industry actors identified in the analysis

Sector	Actors identified	Occurrences (in total)	Occurrences (around the pictogram implementation period)	Occurrences (around the pictogram expansion project)
WINE	**Elected representatives defending vine and wine**			
	Elected representatives (individually)	10	6	4
	Parliamentary Group (National Assembly) “Vine, wine and oenology” (Vigne, vin et œnologie)	1	0	1
	National Association of Wine Elected Officials for Vine and Wine (Association nationale des élus de la vigne et du vin)	1	1	0
	**Trade associations**			
	Wine and Society (Vin et Société)	9	4	5
	Federation of the Great Wines of Bordeaux (Fédération des grands vins de Bordeaux - FGVB)	7	0	7
	Interprofessional Agency for Burgundy Wines (Bureau interprofessionnel des vins de Bourgogne -BIVB)	3	3	0
	Bordeaux and Superior Bordeaux Association (Association Bordeaux et Bordeaux supérieur)	2	0	2
	National Committee of the PDO Wines and Spirits trade association (Comité National des Interprofessions des Vins et Eaux-de vie d'appellation - CNIVE)	2	2	0
	Saône-et-Loire Vine and Wine Federation (Fédération viticole de Saône-et-Loire)	2	2	0

	Sancerroise Wine Union (Union Viticole Sancerroise)	2	2	0
	Interprofessional Committee for Jura wines (Comité Interprofessionnel des vins du Jura - CIVJ)	1	1	0
	Confederation of Burgundy Wine Associations (Confédération des associations viticoles de Bourgogne)	1	1	0
	Confederation of Wine Cooperatives of France (Confédération des coopératives viticoles de France - CCVF)	1	1	0
	French Confederation of Local Wines (Confédération française des vins de pays)	1	1	0
	National Confederation of AOC and AOC Bordeaux and Bordeaux Supérieur wines (Confédération nationale des AOC et des AOC Bordeaux et Bordeaux supérieur)	1	0	1
	Federation for Winemakers of Hérault Cooperatives (Fédération des vignerons coopérateurs de l'Hérault)	1	1	0
	National Interprofessional Wine Agency (Office national interprofessionnel des vins - Onivins)	1	1	0
	Trade union for Côtes de Gascogne vine and wine (Syndicat des Côtes de Gascogne)	1	1	0
	Trade union of the producers of floc de Gascogne (Syndicat des producteurs de floc de Gascogne)	1	1	0
	Trade union for Gard vine and wine (Syndicat des vignerons gardois - SVG)	1	0	1
	Winemakers of France (Vignerons de France)	1	1	0
	Independent Winemakers (Vignerons Indépendants)	1	1	0

	**Individual actors**			
	Winemakers	14	12	2
	Plaimont Producers	2	2	0
	The Lugny estate	1	1	0
	**Others**			
	Wine sector (not specified)	23	1	22
	Sommelier	2	2	0
**SPIRIT**	French Federation for Spirits (Fédération Française des Spiritueux)	3	3	0
**BEER**	Brewers of France (Brasseurs de France)	3	3	0
**SAPRO**	SAPRO "Company and Prevention" (Entreprise et Prévention)	8	8	0
**ALCOHOL SECTOR (in general, not specified)**	Alcohol producers	13	2	11
	Patrick Ricard	1	1	0
**OTHER**	Label printer	1	1	0

APPENDIX 4. Arguments of the AI spread in the French mainstream press from 2000 to 2020

Categories of arguments	Arguments	Number of press articles	Number of occurrences (in total)	Number of occurrences (in period 1)	Number of occurrences (in period 2)
**ARGUMENTS AGAINST THE MEASURE**					
1. A QUESTIONABLE MEASURE					
**EXAGGERATION AND OVERZEALOUSNESS OF ACTORS IN HEALTH**	**EXAGGERATION AND OVERZEALOUSNESS OF ACTORS IN HEALTH**	38	61	19	42
	The pictogram clearly links alcohol to mortality.	21	28	2	26
	This measure is driven by hygiene-first logic.	7	12	2	10
	This measure only serves to reassure the health authorities.	6	7	2	5
	France is one of the only countries to implement the pictogram (along with the USA).	4	5	5	0
	This measure is disproportionate.	3	3	2	1
	This measure opens the floodgates to more virulent messages (“alcohol kills”).	3	3	3	0
	Alcohol (wine) should not be treated in the same way as tobacco.	2	3	3	0
**INEFFECTIVENESS OF THE MEASURE**	**INEFFECTIVENESS OF THE MEASURE**	33	55	24	31
	This measure is ineffective.	20	26	14	12

	Product label will become unreadable (because overcrowded).	11	11	4	7
	This measure is “cosmetic”.	7	8	0	8
	This measure is inappropriate.	7	7	3	4
	The pictogram is not precise enough.	2	2	2	0
	The pictogram is poorly crafted.	1	1	1	0
**MINIMIZATION OF THE SEVERITY OF THE ALCOHOL PROBLEM**	**MINIMIZATION OF THE SEVERITY OF THE ALCOHOL PROBLEM**	10	14	12	2
	Women are already informed and responsible.	6	7	6	1
	Wine is not alcohol or wine is not an alcohol like any other.	5	5	4	1
	FAS remains exceptionally rare.	1	1	1	0
	Alcoholics do not tend use wine.	1	1	1	0
**SKIRTING THE ALCOHOL PROBLEM**	**SKIRTING THE ALCOHOL PROBLEM**	5	5	4	1
	Other warnings should be added to the pictogram.	3	3	3	0
	Other more serious issues (than alcohol) should be addressed.	1	1	1	0
	All human activities are dangerous, so ultimately “living is dangerous”.	1	1	0	1
**OTHER ARGUMENTS**	**OTHER ARGUMENTS**	2	2	2	0
	This measure is ridiculous.	1	1	1	0

	This measure is unpopular.	1	1	1	0
2. COUNTERPRODUCTIVE EFFECTS					
**ON THE ECONOMY**	**ON THE ECONOMY**	40	66	29	36
	This measure attacks the wine sector.	25	43	17	26
	This measure will weaken producers.	11	12	6	6
	The implementation will take producers time.	3	3	3	0
	The cost of implementation will be high.	3	3	1	2
	Bottles carrying the pictogram will be harder to export.	2	2	1	1
	This measure is binding for producers.	1	1	0	1
	This measure will cause a drop in sales (due to of lower consumption).	1	1	1	0
	This measure is unfair for producers if France is the only adopter country (in Europe).	1	1	1	0
**ON WOMEN**	**ON WOMEN**	4	4	3	1
	This measure stigmatizes women and causes guilt.	3	3	3	0
	This measure causes anxiety for women.	1	1	0	1
3. BETTER ALTERNATIVES					
**PROMOTING RESPONSIBLE CONSUMPTION**	**PROMOTING RESPONSIBLE CONSUMPTION AND ITS BENEFITS FOR HEALTH**	13	19	18	1

**AND ITS BENEFITS FOR HEALTH**	Responsible and moderate consumption is not dangerous (even for pregnant women).	9	10	9	1
	Individuals should drink responsibly (individual responsibility).	6	6	6	0
	There are “benefits” of alcohol consumption.	3	3	3	0
**PROMOTING PREVENTIVE PROGRAMS**	**PROMOTING PREVENTIVE PROGRAMS**	12	14	9	5
	Launching wide-reaching education and prevention programs aimed at pregnant women rather than just a simple warning.	8	9	5	4
	Promoting national-scale education on how to responsibly drink wine.	4	5	4	1
**PROMOTING THE ROLE OF HEALTH PROFESSIONALS**	**PROMOTING THE ROLE OF HEALTH PROFESSIONALS**	12	14	12	2
	Prevention is the role of health professionals (not the producers or legislators).	10	11	10	1
	This measure is not the right way to display health information as it must not be construed as a medical prescription.	3	3	2	1
**PROMOTING ALCOHOL-INDUSTRY COLLABORATION ON PREVENTION**	**PROMOTING ALCOHOL-INDUSTRY COLLABORATION ON PREVENTION**	11	14	5	9
	This measure was taken without consulting with business.	6	6	1	5

	The alcohol industry is ready to engage in prevention initiatives.	5	6	2	4
	The alcohol industry proposes to create a “Moderation Council” (a public health–private business partnership).	2	2	2	0
**ARGUMENTS IN FAVOR OF THE MEASURE**					
**IN FAVOR OF IMPLEMENTATION OF THE MEASURE**	**IN FAVOR OF IMPLEMENTATION OF THE MEASURE**	10	12	12	0
	With conditions	2	2	2	0
	Yes, if it is part of a wider concerted effort.	1	1	1	0
	Without conditions	8	10	10	0
	Supportive.	7	8	8	0
	Supportive, and even more committed to spreading the “abstinence during pregnancy” message.	2	2	2	0
**IN FAVOR OF EXPANSION OF THE MEASURE**	**IN FAVOR OF EXPANSION OF THE MEASURE**	16	18	0	18
	With conditions	14	16	0	16
	Yes, to double the size and do more prevention.	7	7	0	7
	Yes, but proposed compromise.	4	4	0	4
	Yes, but maximum size of 8mm.	2	2	0	2
	Yes, but with a grace period before implementation.	2	2	0	2

	Yes, up to double the size.	1	1	0	1
	Without conditions	2	2	0	2
	Supportive.	2	2	0	2
